# First experiences with a diode laser in major gynecological laparoscopic procedures show lack of benefit and impaired feasibility

**DOI:** 10.1007/s10103-022-03696-9

**Published:** 2023-01-05

**Authors:** Saskia Spaich, Sebastian Berlit, Laura Berger, Christel Weiss, Benjamin Tuschy, Marc Sütterlin, Stefan Stefanovic

**Affiliations:** 1 Department of Obstetrics and Gynecology, University Medical Center Mannheim, Heidelberg University, Mannheim, Germany; 2 Department of Medical Statistics and Biomathematics, University Medical Center Mannheim, Heidelberg University, Mannheim, Germany

**Keywords:** Diode laser, Laparoscopy, Hysterectomy, Myoma, Bipolar, Monopolar

## Abstract

**Purpose:**

The aim of this study is to evaluate feasibility and potential benefit of a diode laser in major laparoscopic procedures in gynecology.

**Methods:**

Between 2018 and 2020, a total of 42 cases were enrolled in this study comparing standard electrosurgery with diode laser-supported therapy in laparoscopic supracervical hysterectomy (LASH), total laparoscopic hysterectomy (TLH), or laparoscopic myoma enucleation (LME). Dual wavelength 45 W diode laser light was used to cut and coagulate during laparoscopy in the prospective interventional arm consisting of 11 cases, while 31 matching patients who received conventional treatment with monopolar/bipolar current for the same interventions were retrospectively identified in our laparoscopy database. Recruitment in the prospective interventional laser diode arm was terminated after only 11 patients (instead of planned 50) due to intense hemorrhage and massive smoke development.

**Results:**

A total of 42 cases were analyzed (11 LME, 19 LASH, and 12 TLH). Strong smoke development was evident in all 11 cases in the diode laser arm. It was necessary to convert to bipolar or monopolar current in all hysterectomies (*n* = 9) with initial diode laser implementation due to increased bleeding and smoke development. Conventional current sources had to be used in LMEs (*n* = 2) due to excessive bleeding and poor visibility during enucleation of the fibroid. A significant difference (*p* < 0.0001) was observed regarding smoke development when comparing the laser arm with the control arm.

**Conclusion:**

We found a 45-W diode laser to be inferior to electrosurgical techniques for major laparoscopic gynecologic surgeries regarding bleeding control and smoke development.

## Introduction

Laparoscopic and hysteroscopic procedures are increasingly used to remove individual lesions in the abdominal cavity or even entire organs [1, 2]. Paralleling this development, the fields of electrosurgery, laser, and plasma surgery continue to expand. The techniques and instruments used and further developed in this context are the methods of choice for coagulation and cutting in laparoscopic surgery [3–5]. This is particularly true for operations on bleeding-prone, otherwise sensitive and vital structures.

In electrosurgery, electric current is conducted through the tissue by direct contact. Depending on the number of cutting electrodes used, the current can be monopolar or bipolar. It is important here to ensure that no damage is caused to the patient, surgeon or any implanted devices [1, 2].

In laser surgery, focused light is used to cut through tissue in a very precise way and with little bleeding. Commonly used lasers include the erbium-doped yttrium aluminum garnet (Er:YAG) laser, various diode lasers, the argon laser, the neodymium-doped yttrium aluminum garnet (Nd:YAG) solid-state laser, and the CO2 gas laser. The defined diode laser beam wavelengths of 980 nm and 1470 nm ensure high absorption by water and hemoglobin. Therefore, the thermal penetration depth is significantly lower than with Nd:YAG lasers. This allows safe and more precise laser application in the vicinity of sensitive structures with simultaneous thermal protection of the surrounding tissue. Laser beams can be reflected from surgical instruments located in the surgical field, resulting in injury to adjacent tissue, as well as injury to the surgeon’s eye [6].

Laser-based minimally invasive therapies have already been used for various indications in urology, spine surgery, ear, nose and throat surgery, proctology, and phlebology [7–12]. Mounting data in these fields suggest a reduction of surgery-associated comorbidities, as well as optimization of many relevant clinical aspects in comparison to conventional techniques that favor lasers in minimally invasive procedures [7–12].

Initial experience in gynecology is equally promising and demonstrates that myomas, polyps, uterine septa, cysts, endometriosis, tubal pathologies, peritoneal adhesions, dysplasia, and condylomas can be effectively and successfully treated by laser surgery [13, 14]. Precise and well-controlled cutting with laser light avoids stimulation of the uterine muscles and thus also painful contractions [15]. The coagulation that takes place almost instantly usually ensures a bleeding-free procedure [16].

Some have suggested additional benefits like the possibility of a successful one-trocar LSK adnexectomy, a reduction in pain after removing foci of endometriosis, a reduction in the frequency of reoperations, and higher patient satisfaction with hysteroscopic laser polypectomies [13, 17, 18]. However, with small case numbers and short follow-up intervals, these initial publications lack the statistical power to impact treatment recommendations. Further studies are necessary for a more precise evaluation of the effectiveness, efficacy, and side effect profile of this promising therapeutic method. Furthermore, data on feasibility in more elaborate laparoscopic gynecological procedures such as myoma enucleation and hysterectomy are still lacking despite the promising observations from less complex interventions.

The aim of this study is to generate knowledge regarding the peri- and intraoperative outcome using minimally invasive diode laser in hysterectomies and myoma enucleation since, to the best of our knowledge, there were no preexisting peer reviewed studies on this topic.

## Methods

### Patient population and methodology/study design

This cohort study was conducted at the Department of Gynecology and Obstetrics, University Medical Center Mannheim, Heidelberg University, Mannheim, Germany.

The prospective interventional arm of the study enrolled a total of 11 patients with indication for total laparoscopic hysterectomy (TLH), laparoscopic supracervical hysterectomy (LASH) or laparoscopic myoma enucleation (LME) between 2018 and 2020. Surgery on these 11 patients was performed as indicated with the study causing no interference with regular diagnostics or treatment goals, however with the exception that a diode laser Leonardo® Dual 45 (Biolitec AG, Jena, Germany) was used to cut and coagulate during laparoscopy instead of bipolar current. The diode laser was implemented with a total power setting of 45 W with dual wavelengths—30 W of 980 nm and 15 W of 1470 nm simultaneously in all cases. Following explicit recommendations of the manufacturer, just one fiber type-Bare Fiber 1000 μm Flat Tip (Ref. 503,300,415) was utilized for all indications (TLH, LASH, and LME) for reasons of contact stability of the robust 1000 μm fiber and less tissue debris on the tip upon coagulation when compared to MyoFiber® alternatives.

The patients provided written informed consent. The initial plan to recruit 50 patients in the prospective laser diode arm had to be abandoned because of study termination due to inferiority in terms of smoke development and lack of bleeding control. The matched control group consisted of 31 patients with similar clinical features in whom the same laparoscopic procedures as in the interventional group (TLH, LASH, and LME) were performed by the same gynecological surgeons, only using the standard bi- or monopolar current. These patients were retrospectively identified from our database and served as controls.

Ethics approval for the study incl. usage of retrospective anonymized data was obtained through the Ethics Committee II of Heidelberg University, Medical Faculty Mannheim (2018-600 N-MA, date: September 27, 2018).

### Questionnaire-based evaluation of the laser

Feasibility and potential benefits of the diode laser were evaluated with the use of a questionnaire. To ensure comparability, all patients were operated by the same two expert surgeons with a long-term and similar experience in the field of gynecologic laparoscopic surgery. These two surgeons completed said questionnaire shortly after surgery. They were to report on blood loss (according to suction tube), wavelengths used, laser fiber breakage, laser fiber contact with the treated tissue (i.e., contact mode versus non-contact mode), carbon-dioxide gas consumption, smoke production, and the need of additional electrosurgical instruments (e.g., diathermy) for coagulation and cutting. Furthermore, the surgeons were asked to rate the overall quality of the diode laser instrument, its usability, and suitability for tissue preparation.

### Statistics

A Microsoft Excel 2016 (Microsoft Corporation, Redmond, Seattle, WA, USA) spreadsheet was created to document the responses obtained from the surgeons as well as some objective parameters like surgery duration and blood loss. The data were then imported into the SAS version 9.4 statistics software (SAS Institute, Cary, NC, USA). The quality of the diode laser was compared to that of the standard instruments used in laparoscopic electrosurgery-monopolar and bipolar current-based instruments. We considered the study groups as related. A paired samples *t* test or a Wilcoxon signed rank test were to be performed when comparing continuous variables, depending on the situation. Categorical variables were compared using McNemar’s test and Fisher’s exact test. A repeated measures ANOVA, Friedman’s test or a Cohrane’s *Q* test were to be utilized when comparing more than two groups depending on weather the variable is continuous or categorical and weather the assumptions for parametric tests were met. A *p* value of 0.05 was chosen. If the null hypothesis (no influence of the operation method on the target variable) was rejected when comparing more than two groups, post hoc tests were undertaken between two procedures in each case. If equality of variance could be assumed, a post hoc analysis according to Hochberg was performed. If this was not the case, a post hoc analysis according to Games-Howell was performed.

## Results

Between 2018 and 2020, a total of 42 cases were enrolled in this study comparing standard therapy with diode laser-supported therapy in major gynecological surgery (LASH, TLH, or LME). Diode laser light was used to cut and coagulate during laparoscopy in the prospective interventional arm consisting of 11 cases, while 31 patients who received conventional treatment with mono- and bipolar current as energy source were retrospectively identified and matched in our laparoscopy database, as described in the methods section.

Out of all enrolled 42 patients of the interventional and control groups, 12 received TLH, 19 patients received LASH, and 11 received LME. In total, a diode laser system was used during laparoscopic surgery in 11 women. In the other 31 patients, conventional bi- or monopolar power current was used during laparoscopic surgery.

Patients’ mean age at time of surgery was 45.5 years with no significant differences between groups (Table 1). The average BMI of the patients at time of surgery was 27.4 with no significant differences between groups (*p* = 0.9). In terms of comorbidities, 19% of all patients had pre-existing conditions, again with no significant differences seen between groups (*p* = 0.5). Abdominal surgeries had been performed at some point prior to laparoscopy in 55% of our patient population. There were no significant differences between groups in this respect (*p* = 0.075). The average duration of surgery was 74.3 min—again without significant differences between groups (*p* = 0.9). Finally, no difference in blood loss was observed between the groups (*p* = 0.5). Detailed patient characteristics including age, BMI, surgery duration, loss of blood, previous illnesses, and previous abdominal operations are presented in Table 1.

**Table 1:** Demographic parameters

Variable	All *n* = 42	Diode laser *n* = 11	Bi-/monopolar *n* = 31	Analysis *p* value
TLH: *n* (%) LASH: *n* (%) LME: *n* (%)	12 (29%) 19 (45%) 11 (26%)	2 (18%) 7 (64%) 2 (18%)	10 (32% 12 (39%) 9 (29%)	n.s
Age (years) Mean + / − SD	45.5 + /** − **9	44 + /** − **5	46 + /** − **10	n.s
BMI (kg/m^2^) Mean + / − SD	27.4 + /** − **5.8	25.2 + /** − **5.5	28.1 + /** − **5.8	n.s
Surgery duration (min.) Mean + / − SD	74.3 + /** − **28.0	76.5 + /** − **33.4	73.5 + /** − **26.4	n.s
Loss of blood (ml) Mean + / − SD	91.7 + /** − **117.6	101.8 + /** − **162.9	88.1 + /** − **100.0	n.s
Previous illnesses: *n* (%)				
Yes No	19 (45%) 23 (55%)	6 (55%) 5 (45%)	13 (42%) 18 (58%)	n.s
Previous abdominal surgery: *n* (%)				
Yes No	23 (55%) 19 (45%)	9 (82%) 2 (18%)	14 (45%) 17 (55%)	n.s

*LME* laparoscopic myoma enucleation, *LASH* laparoscopic supracervical hysterectomy, *TLH* total laparoscopic hysterectomy, *BMI* body mass index; *p values based on ANOVA, Kruskal–Wallis test and Fisher´s exact test

Analysis of the questionnaires showed that the diode laser was implemented according to the manufacturer’s recommendations (power setting of 45 W; dual wavelengths of 980 nm and 1470 nm simultaneously) in all cases. The laser fiber was broken in only one case. However, in all cases, intermittent contact between the laser fiber and tissue occurred.

**Fig. 1:**
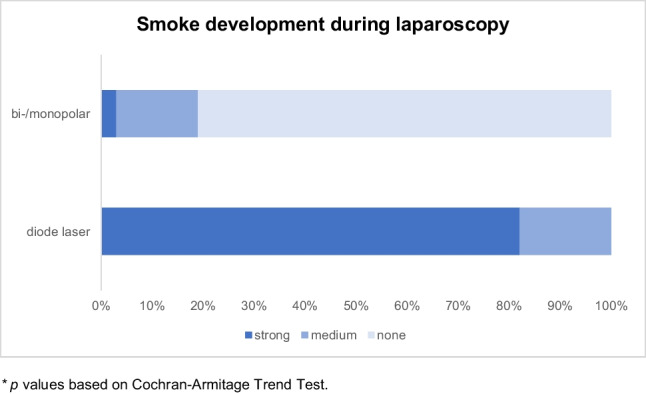
Smoke development during laparoscopy, *p values based on Cochran-Armitage Trend Test

Strikingly, there was very heavy smoke in 9 cases and moderately heavy smoke development in the other two cases, which resulted in the need to switch to conventional electrosurgery several times during all laparoscopies with the diode laser. While the study design mandated the uterine arteries to be coagulated by standard bipolar current, diode laser-facilitated obliteration of the ovarian ligaments was complicated by enhanced bleeding and heavy smoke development leading to poor visibility and repetitive use of bipolar current. Similarly, when the uterus was detached from the vagina in the two TLHs with diode laser application, it was necessary to switch to the conventional current sources because of increased bleeding. In the laparoscopic supracervical hysterectomies, bipolar or monopolar current had to be used additionally when detaching the cervix, in case of parauterine bleeding or occasionally when cutting the tube or the ligamentum rotundum. In the two laparoscopic myoma enucleations, conventional current sources also had to be used due to increased bleeding and poor visibility during enucleation of the myoma. Consistently, a highly significant difference (*p* < 0.0001) in terms of smoke production was observed when comparing the groups (Table 2/Fig. 1).

**Table 2:** Smoke development during laparoscopy

Variable	Diode laser *n* = 11	Bi-/monopolar *n* = 31	Analysis *p* value
Smoke emission: *n* (%)			
--- None --- Medium --- Strong	0 (0%) 2 (18%) 9 (82%)	25 (81%) 5 (16%) 1 (3%)	< 0.0001

^*^p values based on Cochran-Armitage Trend Test

**Fig. 2:**
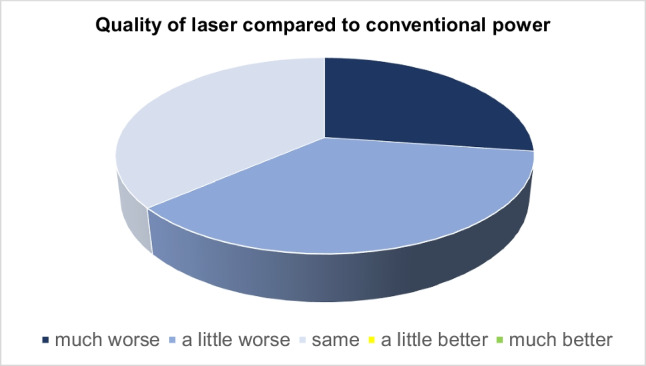
Quality of laser compared to conventional current

In the subjective assessment of the quality of the laser application compared with monopolar or bipolar power, the two operators indicated that quality of laser coagulation was much worse during 3 operations, slightly worse during 4 laparoscopies, and comparable in 4 operations. The ease of use was described as very good or good in 6 cases, and in the other 5 cases no difference was reported compared with the previously used instruments. Preparation for laser surgery was deemed to have gone very well or well in 7 cases. In the other 4 cases, no advantage was seen over bi- or monopolar current in this respect (Fig. 2).

## Discussion

Since minimally invasive surgery has been proven to be advantageous in a number of ways compared to classic surgery when technically feasible, policy makers, patients, hospitals, and surgeons are perpetually searching for a means to improve on the technique and expend its field of application [19]. It is only logical that precise, atraumatic, and hardy instruments capable of efficient bleeding control are highly valued and innovative ways of applying energy to tissue are always being sought. Herein laser surgery seemed to be an excellent modality allowing for much of the advantages of both laparoscopy and precise laser energy to be capitalized on. Diode lasers were particularly interesting in this respect since they were postulated to be excellent when it comes to bleeding control since the energy of their beam is absorbed by blood [20].

Data on diode lasers in particular in laparoscopic use is scarce, while Nd:YAG lasers were studied to a greater extent. Our data shows that the duration of surgery was no different between the groups we studied. Donnez et al. have reported similar duration of myolysis surgery utilizing a Nd:YAG laser [21]. We were unable to find any papers comparing electrosurgery and laser surgery duration in the field of gynecology. We were unable to find any studies that dealt with how laser-based instruments compared to standard electrosurgical techniques when it came to time spent in the OR. The amount of bleeding overall was similar between the groups we studied. It has long been recognized that both laser surgery and electrosurgery allow for quick and efficient hemostasis during surgery [16, 21–23]. However, the surgeon deemed the bleeding to be significantly more pronounced in the surgeries that involved controlling and/or preventing bleeding from major blood vessels. This comes as no surprise since laser hemostasis has been proved problematic when it comes to major bleeding since the energy beam is very focused and the calibrations of the various lasers do not allow for quality cutting, ablation and hemostasis at the same time [24, 25]. Also, no authors noted any significant bleeding when the Nd:YAG laser was employed for myolysis or hysterectomy [26, 27]. Most worrisome is the fact that the use of the diode laser in our study was clearly correlated with excess smoke production. This sometimes obscured the surgeons view so that conversion to standard electrosurgery was necessary. This has been described by some Nd:YAG laser users during fibroma ablation [22].

Overall, the diode laser was found to be on par with standard electrosurgical techniques in only 4 out of 11 surgeries. Nevertheless, the technique might work better for small ablative procedures (e.g. endometriosis ablation). However, the problem of excessive smoke production leading to reduced visibility conditions, which in turn elevates the risk of bleeding until the intraabdominal gas is renewed, remains to be solved by the manufacturer.

### Study limitations

Hospital stay, costs, patient-reported quality of life, and satisfaction with care data as well as long-term follow-up data were not reported on in our study. Our cohort was small due to preterm termination of the study due to inferiority of the interventional laser diode arm, limiting the power of the conclusions of the study. Consequentially, the learning curves of the surgeons had no opportunity to gain dynamics. Finally, various pathologies were treated using the diode laser and the results were analyzed pooling all this data possibly obscuring its true value of the diode laser in treating individual pathologies.

## Conclusion

We have found that a diode laser is equivalent to diathermy when it comes to laparoscopic gynecologic surgery with respect to surgery duration, surgery preparation and ease of use. However, impaired bleeding control was an issue during the most delicate phases (obliteration of major vessels) with an excess of smoke obscuring the surgeon’s visibility conditions. This combination of excessive bleeding and poor visibility due to smoke development was found to be a major drawback and the decisive reason for study termination, thus deeming the dual wavelength diode laser with a power maximum of 45 W unsuitable for performing laparoscopic hysterectomies and myoma enucleations.

## Abbreviations

HE: Hysterectomy
LH: Laparoscopic hysterectomy
LME: Laparoscopic myoma enucleation
TLH: Total laparoscopic hysterectomy
LASH: Laparoscopic supracervical hysterectomy
BMI: Body mass index
